# Frozen Elephant Trunk versus Ascyrus Medical Dissection Stent in Acute Type A Aortic Dissection: Choosing the Appropriate Arch Strategy

**DOI:** 10.1055/a-2915-3346

**Published:** 2026-07-23

**Authors:** Sunil Ohri, Amit Modi, Sanjay Asopa, Geoffrey Tsang

**Affiliations:** Department of Cardiac Surgery 7425 University Hospital Southampton NHS Foundation TrustSouthamptonHampshireUnited Kingdom

**Keywords:** acute type A aortic dissection, frozen elephant trunk, Ascyrus Medical Dissection Stent, aortic arch, malperfusion

## Abstract

Optimal management of the aortic arch during repair of acute type A aortic dissection remains debated. Hemiarch repair can achieve acceptable early survival, but persistent false lumen perfusion and residual distal dissection may contribute to later distal aortic enlargement or reintervention in a subset of patients. The frozen elephant trunk (FET) technique aims to improve distal aortic remodeling by covering intimal tears, expanding the true lumen, and promoting false lumen thrombosis, but it adds operative complexity because it requires more extensive arch reconstruction and supra-aortic vessel management. The Ascyrus Medical Dissection Stent (AMDS), an uncovered nitinol stent, has emerged as an adjunct to hemiarch repair. It is intended to reduce distal anastomotic new entry tears, support true lumen expansion, and facilitate distal aortic stabilization without formal total arch replacement. Contemporary evidence from the DARTS and PERSEVERE studies and observational series suggests that FET and AMDS may serve different operative goals in selected patients, although their roles are overlapping rather than mutually exclusive. FET may be more suitable when more extensive distal arch or proximal descending aortic pathology is present, whereas AMDS may be considered when a less extensive arch procedure is desired in the setting of arch instability or malperfusion. Neither approach should be viewed as universally superior. Instead, strategy should be individualized on the basis of patient age, intimal tear location, malperfusion status, operative risk, and institutional experience. AMDS may represent an additional option in selected high-risk patients.

## Introduction

Acute type A aortic dissection (ATAAD) remains a life-threatening surgical emergency requiring prompt operative treatment. 1 2 Standard repair consists of replacement of the ascending aorta, with or without hemiarch replacement, and resection of the primary entry tear when feasible under hypothermic circulatory arrest. Although this strategy provides acceptable early results in many patients, persistent false lumen perfusion and residual distal dissection may contribute to progressive distal aortic enlargement and the need for secondary aortic intervention during follow-up. 3 4

To address both arch and proximal descending thoracic aortic pathology at the index operation, the frozen elephant trunk (FET) technique has been increasingly adopted in selected patients. 5 6 By combining total arch replacement with antegrade stent grafting of the proximal descending thoracic aorta, FET may promote true lumen expansion, false lumen thrombosis, and more favorable distal aortic remodeling. 7 8 However, these potential advantages must be balanced against the greater complexity of extensive arch reconstruction, including prolonged circulatory arrest, supra-aortic vessel reimplantation, and the possibility of increased neurologic and spinal cord ischemic risk. For this reason, the use of FET in ATAAD is influenced by patient anatomy, clinical presentation, operative risk, and institutional experience.

The Ascyrus Medical Dissection Stent (AMDS; Artivion Inc) has emerged as an adjunct to conventional hemiarch repair. This hybrid prosthesis consists of a proximal polytetrafluoroethylene cuff and a distal uncovered nitinol stent and is deployed in an antegrade fashion across the arch and proximal descending thoracic aorta during open repair. 9 10 The device is intended to reduce distal anastomotic new entry (DANE), support true lumen expansion, and improve proximal aortic remodeling without formal total arch replacement. Because the distal stent is uncovered, branch vessel patency is maintained while structural support is provided to the dissected aorta. Early clinical experience has been encouraging, but the available evidence remains limited and is derived predominantly from single-arm studies and observational series. 11 12

Accordingly, FET and AMDS should be viewed as complementary rather than universally interchangeable strategies. FET provides a more extensive single-stage repair in selected patients with arch and proximal descending thoracic aortic involvement, whereas AMDS may offer a less extensive adjunct to hemiarch repair when total arch replacement is undesirable or higher risk. This review summarizes the contemporary evidence for both approaches and proposes a practical framework for individualized arch strategy selection in ATAAD.

## Methods

A structured narrative review was conducted using MEDLINE and EMBASE databases. Searches combined terms including "acute type A aortic dissection," "frozen elephant trunk," "Ascyrus Medical Dissection Stent," "AMDS," "aortic arch," "malperfusion," and "aortic remodeling." Publications from January 2000 through January 2025 were reviewed. Cohort studies, registry analyses, prospective trials, and systematic reviews were included. Outcomes of interest included 30-day and in-hospital mortality, neurologic injury, spinal cord ischemia, DANE incidence, false lumen thrombosis, aortic remodeling, and reintervention rates. Because of significant heterogeneity among available studies, formal quantitative meta-analysis was not performed. Findings were synthesized with a focus on operative decision-making.

### Pathophysiology of Distal Aortic Failure

A major determinant of adverse distal aortic remodeling after ATAAD repair is persistent false lumen patency and pressurization in the descending thoracic aorta. 3 13 Two related mechanisms may contribute to downstream aortic events:

1. Residual distal tears and DANE: persistent communication between the true and false lumens, including tears at the distal anastomosis after hemiarch repair, can maintain false lumen pressurization and promote progressive distal aortic enlargement and later reintervention. 14
2. True lumen compression and dynamic malperfusion: in some patients, false lumen pressurization may compress the true lumen and impair distal branch vessel perfusion, contributing to visceral, renal, spinal, or peripheral malperfusion. This mechanism is particularly relevant in the acute setting and may coexist with adverse distal aortic remodeling. 15 16

FET addresses persistent false-lumen pressurization by covering intimal tears in the arch and proximal descending thoracic aorta, promoting true-lumen expansion and false-lumen thrombosis within the stented segment. In selected patients, this may also improve distal malperfusion. AMDS, used as an adjunct to hemiarch repair, is intended to reduce DANE, support true-lumen expansion, and improve proximal arch remodeling without formal total arch replacement. Thus, both strategies may influence false-lumen pressurization and malperfusion, although they do so with different operative extent and complexity.

### Frozen Elephant Trunk

#### Operative Technique and Rationale

The FET procedure combines total arch replacement with antegrade deployment of a hybrid stent graft into the proximal descending thoracic aorta under hypothermic circulatory arrest and selective antegrade cerebral perfusion. 5 6 Depending on the prosthesis and operative strategy, the supra-aortic vessels may be managed with individual branch reconstruction or island reimplantation. Currently used FET prostheses include Thoraflex Hybrid (Terumo Aortic), E-vita Open Neo (Artivion), Cronus (MicroPort), and FROZENIX/J Graft FROZENIX (Japan Lifeline/JOTEC lineage), although device availability varies by region, and Thoraflex and E-vita are the most widely reported in the contemporary literature. The distal anastomosis is commonly performed in zone 2, and the stented portion extends into the proximal descending thoracic aorta. This strategy is intended to exclude arch pathology, cover residual intimal tears in the arch or proximal descending thoracic aorta, promote true lumen expansion and false lumen thrombosis within the stented segment, and provide a proximal landing zone for secondary endovascular intervention when required.

#### Contemporary Evidence

A 2024 systematic review and meta-analysis by Naito and Takagi encompassing 21 nonrandomized studies and 3,240 patients demonstrated that FET was associated with significantly lower short-term mortality compared with conventional repair (odds ratio: 0.58; 95% confidence interval [CI]: 0.44–0.78; *p* < 0.01). 7 In the subgroup of ATAAD patients, FET was associated with reduced all-cause mortality (hazard ratio [HR]: 0.55; 95% CI: 0.39–0.79) and lower aortic reintervention (HR: 0.62; 95% CI: 0.39–0.99) over medium-term follow-up. A separate meta-analysis of 35 studies including 3,211 patients undergoing FET for ATAAD reported pooled rates of in-hospital mortality, postoperative stroke, and spinal cord injury of 7%, 5%, and 3%, respectively. 8

FET produces high rates of false lumen thrombosis at the stented segment (exceeding 90%), with thrombosis extending to the mid and distal descending aorta in 34 to 72% of cases at midterm follow-up. 17 18 Importantly, early mortality following FET is strongly influenced by preoperative patient condition, particularly in complicated ATAAD with malperfusion, rupture, or preoperative hemodynamic instability, where 30-day mortality may exceed 40%. 19

The 2024 EACTS/STS (European Association for Cardio-Thoracic Surgery/Society of Thoracic Surgeons) Guidelines for diagnosing and treating acute and chronic syndromes of the aortic organ 20 recognizes FET as an appropriate technique in selected patients, noting an individualized approach to arch management in ATAAD. It recommends a tear-oriented strategy and mentions that in patients with entry tears in the distal arch or proximal descending aorta, implantation of an FET may be considered in specialized centers.

#### Advantages and Limitations of Frozen Elephant Trunk

Potential advantages of FET include more extensive treatment of arch and proximal descending aortic pathology at the index operation, promotion of true-lumen expansion and false-lumen thrombosis, and creation of a proximal landing zone for future thoracic endovascular intervention, if required. Observational studies suggest that these features may translate into improved downstream aortic remodeling and lower rates of later aortic reintervention in selected patients. These benefits are most pronounced in younger patients with extensive dissection, arch or proximal descending intimal tears, connective tissue disorders, or preexisting arch aneurysm.

These benefits must be balanced against the greater technical demands of total arch replacement, including longer circulatory arrest and cerebral perfusion times. Reported pooled rates after FET in ATAAD are approximately 5% for stroke and 3% for spinal cord injury. Accordingly, FET is best considered in the context of anatomical extent, intimal tear location, malperfusion profile, operative risk, and institutional experience, rather than as a universally preferred strategy.

### Ascyrus Medical Dissection Stent

#### Device Description and Operative Technique

The AMDS is a hybrid prosthesis consisting of a proximal polytetrafluoroethylene cuff and a distal uncovered self-expanding nitinol stent that extends into the aortic arch and proximal descending thoracic aorta. 9 10 During hypothermic circulatory arrest, the device is introduced in an antegrade fashion into the true lumen after transection of the ascending aorta, typically with a short segment of native aorta preserved proximal to the innominate artery to accommodate the cuff. The proximal cuff is then secured to the native aortic wall, and the ascending graft is anastomosed to the cuff to complete the repair. Because the distal stent is uncovered, branch vessel patency is preserved and formal supra-aortic vessel reimplantation is not required. Deployment of AMDS adds approximately 10 minutes to operative time compared with isolated hemiarch repair.

#### Clinical Evidence: DARTS Observational Study

The Dissected Aorta Repair Through Stent Implantation (DARTS) observational study is the primary prospective study evaluating AMDS. This multicenter, nonrandomized trial enrolled 47 consecutive patients with acute DeBakey type I dissection at five Canadian and one German center (March 2017–January 2019). 9 Three-year outcomes demonstrated that AMDS implantation was safe and reproducible, with 30-day mortality of 13.0% and 1-year mortality of 19.6% in this critically ill population, including 56.5% with preoperative malperfusion. 21 22 Crucially, no DANE tears were detected in any AMDS-treated patient throughout 3 years of follow-up. This is a significant finding given the high DANE incidence reported with isolated hemiarch repair. 11 True lumen diameter remained stable or increased in 100% of patients at 1 year, with positive arch remodeling in all patients and favorable proximal descending remodeling in 77.1%.

#### Clinical Evidence: PERSEVERE Trial

The PERSEVERE US IDE trial (NCT05174767) was conducted to support the Food and Drug Administration (FDA) Pre-Market Approval. One-year outcomes presented at the 61st STS Annual Meeting (January 2025) across 93 patients demonstrated a 72% reduction in all-cause mortality and a 54% reduction in primary major adverse events compared with historical hemiarch controls at 30 days. 23 24 DANE tears were absent in all AMDS-treated patients at 1 year. The need for unanticipated aortic reoperation was 4.3%, comparing favorably with published reintervention rates after standard hemiarch repair. These data supported FDA approval of AMDS under a Humanitarian Device Exemption.

#### Contemporary Observational Evidence

Luehr et al 25 reported outcomes in 57 consecutive ATAAD patients treated with AMDS at two German centers (2020–2022). Actual in-hospital mortality (16%) was substantially lower than predicted risk by the German Registry score (22%), supporting the safety profile of AMDS in real-world practice. A notable AMDS-specific complication, central stent collapse, was identified in 9% ( *n* = 5), underscoring the importance of appropriate device sizing and anatomic selection to avoid gothic arch configurations and excessive aortic diameters. White et al 12 demonstrated in a propensity-matched comparison ( *n* = 114) that AMDS significantly reduced DANE incidence compared with isolated hemiarch repair (11.8 vs. 43.3%; *p* = 0.002), with favorable false lumen thrombosis rates in the arch, proximal descending aorta, and at the pulmonary artery bifurcation level.

Pitts et al 10 provided a comprehensive 2024 review summarizing AMDS indications, technique, and pitfalls, emphasizing that careful preoperative anatomic evaluation, including assessment for gothic arch morphology, preexisting arch aneurysm, and reentry tears in the arch is essential to avoid device failure. A multicenter study by Cabrucci et al, 26 encompassing 46 consecutive patients across European and US institutions from 2021 to 2025, reported that AMDS implantation was effective, with favorable early survival linked to moderate hypothermia and optimized cerebral perfusion strategies.

The long-term durability of AMDS remains incompletely defined. Case reports have documented redo aortic arch interventions in patients previously treated with AMDS, driven by persistent false lumen patency and distal reentry tears, underscoring the need for vigilant lifelong surveillance after AMDS implantation and a low threshold for secondary intervention. 27 These findings align with series demonstrating that late aortic failure and reoperation are not uncommon after proximal-only repair of acute type A dissection, with patent false lumen representing the dominant driver of downstream aortic events and reintervention. 28 29

#### Advantages and Limitations of Ascyrus Medical Dissection Stent

**Fig. 1:**
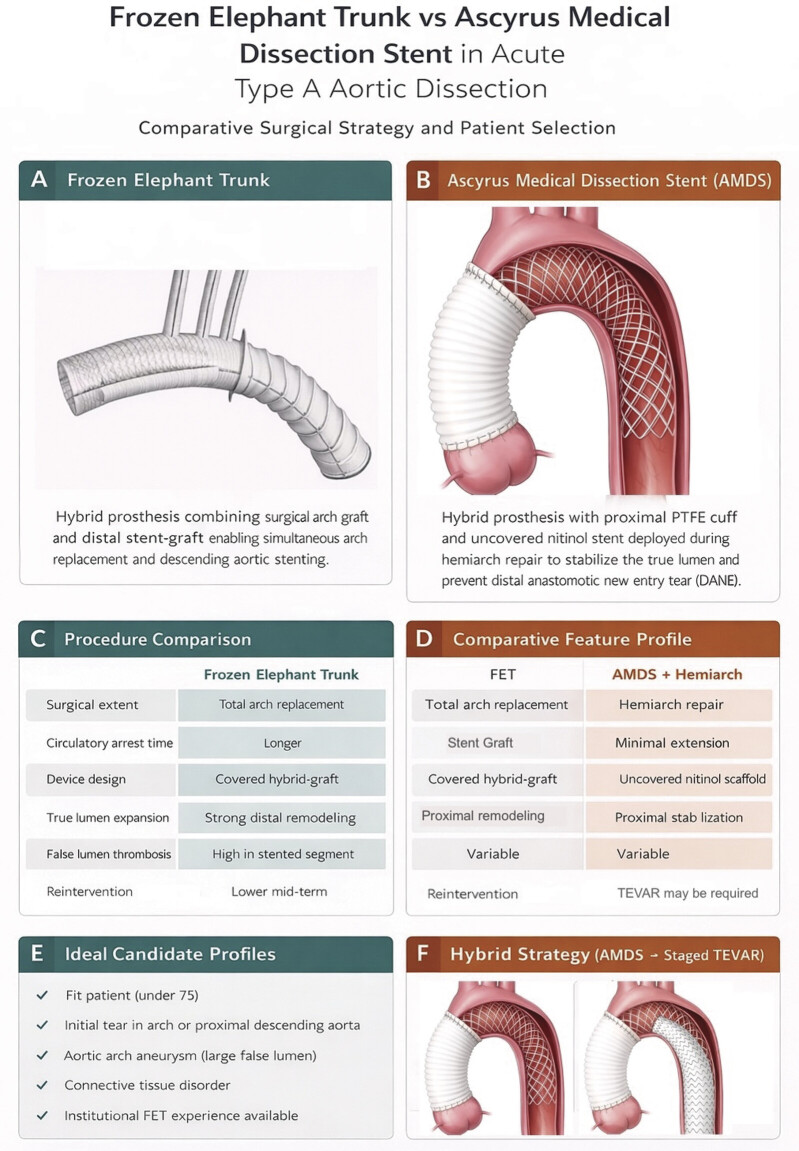
Schematic comparison of frozen elephant trunk and Ascyrus Medical Dissection Stent strategies in acute type A aortic dissection, illustrating key operative characteristics, risk profiles, aortic remodeling targets, and complementary clinical roles. TEVAR, thoracic endovascular aneurysm repair. ( **A** ) FET hybrid prosthesis; ( **B** ) AMDS hybrid prosthesis (proximal PTFE cuff + uncovered nitinol stent); ( **C** ) procedure comparison; ( **D** ) comparative feature profile (FET vs AMDS + hemiarch); ( **E** ) ideal candidate profiles; ( **F** ) staged hybrid strategy (AMDS → staged TEVAR).

AMDS offers several operative advantages: minimal additional circulatory arrest time, avoidance of supra-aortic head and neck vessel reimplantation, proximalization of distal aortic anastomosis, rapid restoration of branch vessel perfusion, and applicability in elderly or hemodynamically unstable patients. Its primary limitations include the inability to exclude intimal tears in the aortic arch and proximal descending aorta, uncertain long-term aortic remodeling of the mid and distal descending aorta, and the risk of stent collapse in anatomically unsuitable patients ( Table 1 , Fig. 1 ).

**Table 1:** Comparative characteristics of frozen elephant trunk and Ascyrus Medical Dissection Stent for acute type A aortic dissection

Feature	Frozen elephant trunk	Ascyrus Medical Dissection Stent
Operative addition	Major (total arch replacement)	Minor (∼10 min)
Circulatory arrest	Prolonged	Minimally extended
Stroke risk	Pooled FET series 5%; comparative studies do not clearly prove a higher stroke risk than hemiarch	Similar to hemiarch
Spinal cord risk	Present (2–5%)	Minimal
DANE prevention	Yes (arch covered)	Yes (all patients, trials)
False lumen thrombosis	High (>90% at stent level)	Proximal arch only
Distal remodeling	Superior (midterm)	Limited evidence
Late reintervention	Reduced	Potentially required
Best candidates	Young; arch/proximal descending tear; CTD; arch aneurysm	Malperfusion; elderly; hemodynamic instability; ascending tear

Abbreviations: CTD, connective tissue disorder; DANE, distal anastomotic new entry; FET, frozen elephant trunk.

#### Neurologic Complications

Neurologic injury is a principal driver of morbidity and mortality in ATAAD surgery. The pooled stroke rate for FET across major series approximates 5%, with spinal cord ischemia in 2 to 5%. 7 8 The duration of hypothermic circulatory arrest is a significant independent predictor of both adverse neurologic outcomes, reinforcing the importance of operative efficiency. 30

AMDS, by avoiding arch vessel reimplantation and minimizing additional circulatory arrest time, carries a neurologic risk profile comparable to isolated hemiarch repair. This is a meaningful advantage in elderly patients, those with preexisting cerebrovascular disease, or in situations where speed of surgery is critical to rescue from preoperative malperfusion.

#### Patient Selection Framework

**Fig. 2:**
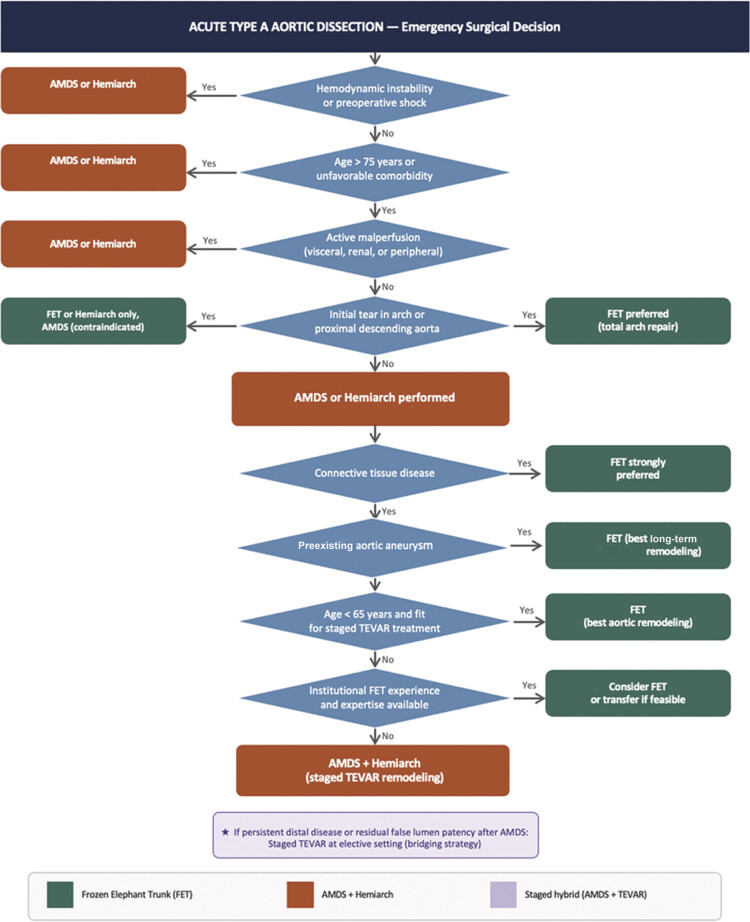
Decision-making algorithm for aortic arch strategy in acute type A aortic dissection. A framework integrating patient risk profile, intimal tear location, malperfusion status, and arch anatomy to guide individualized selection between frozen elephant trunk and hemiarch repair with Ascyrus Medical Dissection Stent (AMDS). AMDS, Ascyrus Medical Dissection Stent; FET, Frozen Elephant Trunk; TEA, Thoracic Endovascular Aorta; TEVAR, Thoracic Endovascular Aortic Repair.

A practical decision-making framework should integrate clinical, anatomical, and operative factors ( Table 2 , Fig. 2 ).

**Table 2:** Proposed patient selection framework for arch strategy in acute type A aortic dissection

Clinical scenario	Preferred strategy
Young patient (<65 y) with arch or proximal descending intimal tear	Frozen elephant trunk
Connective tissue disorder (Marfan, Loeys–Dietz)	Frozen elephant trunk
Pre-existing arch aneurysm (>45 mm)	Frozen elephant trunk
Ascending aorta primary tear; no arch aneurysm	Frozen elephant trunk or AMDS or hemiarch
Active malperfusion (visceral, renal, peripheral)	AMDS or hemiarch
Elderly patient (>75 y) or significant comorbidity	AMDS or hemiarch
Hemodynamic instability; preoperative shock	AMDS or hemiarch
Gothic arch morphology or large aortic diameter at arch	Frozen elephant trunk or hemiarch only (AMDS contraindicated)
Failed AMDS or persistent distal disease after stabilization	Frozen elephant trunk with staged TEVAR

Abbreviations: AMDS, Ascyrus Medical Dissection Stent; TEVAR, thoracic endovascular aneurysm repair.

#### Staged and Hybrid Approaches

An emerging concept is the use of AMDS as a bridge to staged thoracic endovascular aneurysm repair (TEVAR) rather than a definitive single-stage repair. By stabilizing the proximal arch, resolving malperfusion, and eliminating DANE tears, AMDS may enable elective TEVAR of residual distal disease under optimal conditions, avoiding the combined operative burden of total arch replacement and descending aortic stenting in a single emergency setting. 10 The preservation of the native arch vessels, a design feature of AMDS, facilitates subsequent minimally invasive reintervention options.

The DARTS remodeling substudy demonstrated that patients with greater numbers of false lumen communications to branch vessels were more likely to experience distal aortic growth, particularly at zone 3, identifying a group potentially requiring secondary intervention after AMDS stabilization. 31 These findings underscore the importance of systematic follow-up imaging after AMDS deployment.

## Limitations of Current Evidence

The existing literature on both FET and AMDS consists predominantly of observational series, single-center cohorts, and registry studies with limited follow-up durations. Direct randomized comparison of FET and AMDS is lacking and ethically challenging given the emergency nature of ATAAD surgery. Substantial selection bias is inherent, as surgeons choose strategy based on patient condition, anatomy, and institutional experience. The only prospective trial data for AMDS are derived from the DARTS and PERSEVERE studies with intermediate follow-up. Long-term (>5 years) aortic remodeling data for AMDS remain awaited.

## Conclusion

Management of the aortic arch during ATAAD repair should be individualized, depending on the clinical status of the patient. FET and AMDS are not competing technologies but complementary strategies addressing different aspects of dissection pathophysiology.

To date, FET offers superior long-term aortic remodeling and reduced late reintervention, benefits most realized in younger patients with arch intimal tears, connective tissue disease, or preexisting arch aneurysm who can tolerate the physiological demands of total arch replacement. AMDS offers a simpler, faster, and less neurologically hazardous approach that resolves malperfusion, eliminates DANE tears, and is better suited to elderly, unstable, or high-risk patients with ascending aortic tears and without arch aneurysm. Both strategies are underpinned by an evolving evidence base, and the staged hybrid approach, AMDS stabilization followed by selective TEVAR, represents a promising evolving strategy. Future prospective registries and longer-term follow-up are needed to better define the durability of AMDS and to identify which patients ultimately require secondary intervention.
